# Forum shifting in global health security

**DOI:** 10.2471/BLT.23.290480

**Published:** 2023-10-31

**Authors:** Clare Wenham

**Affiliations:** a Department of Health Policy, London School of Economics and Political Science, Houghton Street, London, WC2A 2AE, England.

## Abstract

Global health security is an increasingly complex regime. The failures of global governance and norms of cooperation during the coronavirus disease 2019 (COVID-19) pandemic and the re-entrenchment to nationalist policy-making have created impetus for new governance arrangements, institutions and policy development. These changes include amendments to the *International health regulations* (IHR), development of a pandemic convention or accord, convening of the High-Level Meeting on Pandemic Preparedness and Response, establishment of the Pandemic fund, and development of the medical countermeasures platform, among others. These various developments claim to be in synergy with each other, but understanding of regime complexes and forum shifting from international relations reveal the power dynamics which underlie these processes. I use these concepts to demonstrate how states are transferring negotiations from one institutional location to another in search of more favourable outcomes, or are creating strategic uncertainty within negotiations to avoid future accountability. I further highlight three risks posed by these developments: (i) an increasingly complex landscape for global health security; (ii) erosion of the World Health Organization’s authority in global health security; and (iii) dominance of high-income state positions within these negotiations.

## Résumé

La sécurité sanitaire mondiale est un système de plus en plus complexe. Les échecs essuyés par la gouvernance mondiale et les normes en matière de coopération durant la pandémie de maladie à coronavirus 2019 (COVID-19), ainsi que le retour à une élaboration nationaliste des politiques, ont entraîné la création de mécanismes de gouvernance, d'institutions et de programmes politiques inédits. Ces transformations se traduisent notamment par des amendements au Règlement sanitaire international (RSI), la mise au point d'une convention ou d'un accord face aux pandémies, l'organisation de la Réunion de haut niveau inédite sur la prévention, la préparation et la riposte face aux pandémies, l'établissement du Fonds de lutte contre les pandémies, mais aussi l'instauration de la plateforme de contre-mesures médicales. Ces différents changements affirment œuvrer en synergie, mais la compréhension des complexités du système et l'évolution des débats, qui se détachent des relations internationales, révèlent les dynamiques de pouvoir qui sous-tendent ces processus. J'utilise ces concepts pour montrer comment les États transfèrent les négociations d'un siège institutionnel à l'autre en quête de résultats plus favorables, ou créent une incertitude stratégique dans le cadre des négociations pour se soustraire à de futures responsabilités. Je souligne également trois risques que comportent ces changements: (i) un paysage sans cesse plus complexe pour la sécurité sanitaire mondiale; (ii) l'érosion de l'autorité exercée par l'Organisation mondiale de la Santé en la matière; et enfin, (iii) la position dominante des États à revenu élevé dans les négociations.

## Resumen

La seguridad sanitaria mundial es un sistema cada vez más complejo. Los fracasos de la gobernanza mundial y de las normas de cooperación durante la pandemia de la enfermedad por coronavirus de 2019 (COVID-19) y el repliegue hacia la formulación de políticas nacionalistas han impulsado nuevos acuerdos de gobernanza, instituciones y desarrollo de políticas. Estos cambios incluyen enmiendas al Reglamento Sanitario Internacional (RSI), el desarrollo de una convención o acuerdo sobre pandemias, la convocatoria de la Reunión de Alto Nivel sobre Preparación y Respuesta ante Pandemias, el establecimiento del Fondo para Pandemias y el desarrollo de la plataforma de contramedidas médicas, entre otros. Estos diversos desarrollos pretenden funcionar en sinergia, pero la comprensión de las complejidades del sistema y la evolución de los debates, al margen de las relaciones internacionales, revelan la dinámica de poder que subyace a estos procesos. Utilizo estos conceptos para demostrar cómo los Estados trasladan las negociaciones de un lugar institucional a otro en busca de resultados más favorables o crean incertidumbre estratégica dentro de las negociaciones para evadir futuras responsabilidades. Además, destaco tres riesgos que plantean estos acontecimientos: (i) un panorama cada vez más complejo para la seguridad sanitaria mundial; (ii) la pérdida de autoridad de la Organización Mundial de la Salud en materia de seguridad sanitaria mundial; y (iii) el predominio de las posiciones de los Estados de ingresos altos en estas negociaciones.

## ملخص

إن الأمن الصحي العالمي هو نظام معقد على نحو متزايد. لقد أدت إخفاقات الحوكمة العالمية، ومعايير التعاون خلال جائحة مرض فيروس كورونا 2019 (كوفيد 19)، وإعادة ترسيخ عملية صنع السياسات القومية، إلى خلق زخم لترتيبات حوكمة جديدة، ومؤسسات، وتطوير للسياسة. وتشمل هذه التغييرات تعديلات في اللوائح الصحية الدولية (IHR)، وتطوير اتفاقية أو اتفاق بشأن الجائحة، وعقد اجتماع رفيع المستوى بخصوص الاستعداد والاستجابة للجوائح، وإنشاء صندوق للأوبئة، وتطوير منصة للتدابير الطبية المضادة، فضلًا عن أمور أخرى. تزعم هذه التطورات المختلفة أنها متضافرة مع بعضها البعض، إلا أن فهم تعقيدات النظام وتحول المنتدى من العلاقات الدولية، يكشف عن ديناميكيات القوة التي تكمن وراء هذه العمليات. أنا أستخدم هذه المفاهيم لتوضيح كيف تقوم الدول بنقل المفاوضات من موقع مؤسسي إلى آخر، بحثًا عن نتائج أكثر ملاءمة، أو تقوم بخلق حالة من عدم اليقين الاستراتيجي في تفاصيل المفاوضات لتجنب المساءلة في المستقبل. كما أقوم بتسليط الضوء كذلك على ثلاثة مخاطر تفرضها هذه التطورات: (1) المشهد متزايد التعقيد للأمن الصحي العالمي؛ و(2) تآكل سلطة منظمة الصحة العالمية في الأمن الصحي العالمي؛ و(3) سيطرة المناصب الحكومية ذات الدخل المرتفع في هذه المفاوضات.

## 摘要

全球卫生安全形势日益复杂。新型冠状病毒肺炎 (COVID-19) 疫情期间，全球治理和合作规范方面的失败以及民族主义政策的重新制定，推动了新的治理安排、机构的确立和新政策的制定。这些变化包括修订《国际卫生条例》(IHR)、制定大流行病公约或条约、召开流行病预防和应对高层会议、设立流行病基金以及开发医疗对策平台等。以上各项事态的发展可以说是相互协同的，但理解治理体系的复杂性和基于国际关系的论坛场所转变揭示了这些进程背后的权力动力学。我用这些概念来说明各国是如何将谈判从一个机构地点转移到另一个机构地点，以寻求更有利的结果，或者如何在谈判中建立战略不确定性，从而避免未来被问责。此外，我还特别说明了这些事态发展带来的三个风险：(i) 全球卫生安全形势日益复杂；(ii) 世界卫生组织在全球卫生安全方面的权威逐渐丧失；(iii) 高收入国家在这些谈判中占主导地位。

## Резюме

Международная безопасность в области здравоохранения представляет собой постоянно усложняющуюся систему управления. Неудачи международного управления и нарушения норм сотрудничества во время пандемии коронавирусной инфекции 2019 года (COVID-19) и возврат к формированию национально ориентированной политики послужили стимулом для создания новых механизмов управления, институтов и разработки правил. К числу таких изменений относятся: внесение изменений в Международные медико-санитарные правила (ММСП), разработка конвенции или соглашения по борьбе с пандемией, созыв заседания высокого уровня по готовности к пандемии и ответным мерам, создание Фонда по борьбе с пандемией, разработка платформы для медицинского обеспечения и др. Эти различные изменения предполагают взаимное усиление друг друга, однако понимание сложностей систем управления и изменений дискуссионных площадок в международных отношениях позволяет выявить лежащую в основе этих процессов динамику влияния. Использование этих концепций позволяет продемонстрировать, как государства переносят переговоры из одного институционального пространства в другое в поисках более благоприятного исхода или создают стратегическую неопределенность в рамках переговоров во избежание ответственности в будущем. Далее приводятся три риска, порождаемые этими событиями: (i) все более сложная обстановка с точки зрения международной безопасности в области здравоохранения; (ii) подрыв авторитета Всемирной организации здравоохранения по вопросам международной безопасности в области здравоохранения; (iii) доминирование позиций государств с высоким уровнем дохода на этих переговорах.

## Introduction

Global health security, defined by the World Health Organization (WHO) as “the activities required, both proactive and reactive, to minimize the danger and impact of acute public health events that endanger people’s health across geographical regions and international boundaries,” is increasingly complex.^1^

Traditionally, WHO has been at the centre of global health security activities, as the international agency with the mandate “to act as the directing and coordinating authority on international health work.”^2^ WHO’s current authority for global health security derives from the *International health regulations* (IHR), 2005.^3^ This authority includes: (i) requiring states to have certain core capacities in public health to be able to prevent, detect and respond to an emerging infectious disease; (ii) designating an event a public health emergency of international concern and in doing so issuing temporary recommendations to states, including on travel and trade, to minimize the potential effects of the pathogen; (iii) allowing anyone to report a disease to WHO, a prerogative which was previously reserved only for states, although States Parties concerned need to verify the status;^3^ and (iv) taking an all-risk approach to disease. Thus, WHO has the role of global health security coordinator.^4^ However, perceived failures with recent health emergencies, such as a failure to follow temporary recommendations of the IHR, have meant that the IHR and WHO’s authority has been challenged^5^^–^^7^ and, in parallel, new mechanisms and institutions are being developed that add to the governance landscape of global health security. These bodies include the World Bank, International Monetary Fund, World Trade Organization (WTO), United Nations (UN) General Assembly, philanthropic foundations and nongovernmental organizations.

In this paper, I use the concepts of regime complexes and forum shifting from the field of international relations to demonstrate that current global health security governance is increasingly a regime complex. The current governance is characterized by a multitude of actors, institutions and processes engaged in tackling global health challenges.^8^^–^^12^ Arguably, such an arrangement has come about because of several factors, including: (i) increased globalization causing the transnational spread of disease and products that are harmful to health, each of which requires more complex international governance arrangements; (ii) the normalization of multilateral governance in all spheres; (iii) enhanced scientific and medical practice and learning; and (iv) a so-called catalytic trigger, such as an epidemic or pandemic, which precipitates new governance practices.^10^^–^^12^

However, within the regime complex of global health security, states are still the driving force behind these arrangements, despite most policy development occurring within international organizations. However, this dominance of states leads to increased inequality between states within multilateral negotiations and processes. This situation arises because: (i) the regime complex creates multiple processes which states must navigate and some states have less capacity to do so; and (ii) states engage in forum shifting, to move issues between institutions and actors to gain more favourable outcomes. We are currently witnessing an important shift in the evolving global health security regime to new forums. Moreover, given that high-income states have influence in the regime complex of global health security,^13^ the institutions involved in this area may face diminished authority. This situation has potential implications for the normative power of such institutions, particularly WHO; the way stakeholders must navigate this landscape; and ultimately meaningful global health security, which these evolving power dynamics may jeopardize.

## Regime complex

A regime complex refers to “an array of partially overlapping and non-hierarchical institutions that includes more than one international agreement or authority. The institutions and agreements may be functionally or territorially defined. Regime complexity refers to international political systems of global governance that emerge because of the coexistence of rule density and regime complexes.”^14^

The concept of regime complexes has been increasingly used to understand the diverse and interconnected set of rules, norms and institutions that govern global health,^11^^,^^15^ including states, intergovernmental organizations, nongovernmental organizations, philanthropic foundations, civil society groups, private sector entities and academic institutions.^10^ These actors engage in various forms of collaboration, cooperation and competition through international law and norm setting to address pressing global health issues.^15^ The coexistence of multiple regimes within the global health governance landscape creates both opportunities and challenges. On the one hand, a regime complex can foster the sharing of knowledge, resources and best practices across different sectors. On the other hand, the complexity and overlapping nature of regimes can lead to fragmentation, duplication and coordination challenges.

Within global health, subregime complexes also exist including antimicrobial resistance,^16^ donor health programmes,^17^ donor funding,^18^ pharmaceutical production^19^ and global health security.^11^ The global health security regime has been defined as “the implicit or explicit principles, norms, rules and decision-making procedures by which international actors (including both states and civil society organizations) aim to protect their constituencies from the transmission of diseases from one area to another.”^11^ Based on an historical analysis of the development of the global health security regime, four key periods have been proposed: the unilateral quarantine regime in the early modern period (1500–1800); the nascent sanitary conference regime starting in the 19th century; the institutionalized sanitary coordination regime at the start of the 20th century; and the health cooperation regime starting with the founding of WHO in 1948.^11^

WHO achieved success in its initial decades, both by consolidating regional governance mechanisms,^20^ and through notable achievements such as the eradication of smallpox.^21^ However, the emergence of many new actors and challenges to its legitimacy that arose from the Ebola virus disease outbreaks and the coronavirus disease 2019 (COVID-19) pandemic^9^^,^^22^ suggest that WHO may no longer be seen as the primary authority within the global health security regime. This paper suggests that the catalytic trigger for a crisis of authority is COVID-19.^23^ Given this scenario, there may be four future trajectories: (i) greater authority for WHO; (ii) dominance by a concert of powers; (iii) global rebalancing of power among states; and (iv) civil society leadership.^11^ In the wake of the multiple failures in dealing with COVID-19 in many domestic and international governance settings, numerous calls have been made for the need to reimagine multilateralism to make it fit for purpose^15^ – the questions are: for whose purpose,^24^ and who is making the calls for new governance arrangements? Such changes will have very real effects on what the global health security regime will look like in the future.

## Forum shifting

Given the proliferation of actors in the regime complex for global health security, selecting an institutional location for governance and transforming this location to serve particular interests has become a key feature of policy-making.^25^ Forum shifting refers to the strategic process through which actors move the location of negotiations and discussions to forums or institutions which better meet their needs. The purpose of doing so can be multiple, such as: to seek more favourable outcomes; to relieve growing political pressure within other forums; to create competing norms to challenge the dominant discussions elsewhere; or to promote integration across different regimes.^26^

To optimize authority, and ultimately outcomes, in negotiations, states need to consider several factors including: membership; mandate; decision-making processes; enforcement options; organizational culture and historical development; secretariat capacity; funding arrangements; provisions for reservations to be lodged; arrangements for provision of technical or scientific advice; provisions for withdrawal; and links with other forums of governance.^27^ In this way, high-income countries might move negotiations to a forum where decision-making is linked to material contribution to have a greater opportunity to shape the outcome of the policy decision. Similarly, blocks of low- and middle-income states might push to move an issue into the UN system where collectively, through group coalitions, they would have more votes in a one-state one-vote model. As a result, forum shifting has profound implications for global governance and affects the distribution of power, effectiveness of decision-making, and overall coherence of policies and actions. Indeed, in effect, forum shifting can be considered to result in negotiations never really being completed.^28^ When a conclusion may be reached in one forum, if it is not to the interests of all involved, then a new process will emerge or the negotiations will continue in another forum. Thus, forum shifting is not only a policy development tactic, but also a manifestation of power, particularly if it is done in the absence of material power.^29^

## Examples of forum shifting

For some Member States, WHO and the IHR, even if imperfect, remain the core of the regime complex of global health security. These states have proposed amendments to be made to the IHR to enhance their operability and implementation. This move is being led by a working group for the IHR under the auspices of WHO. This process is centred on reviewing and redrafting legal text to improve the IHR and make them fit for purpose in the post-COVID-19 era. This approach was initially proposed by the United States of America (USA),^30^ which considered the IHR a forum for governing global health security^31^ because the regulations are technical public health policies^32^ and they improve surveillance,^33^^,^^34^ but they do not challenge sovereign decision-making.^6^

At the same time, the European Union (EU) proposed a new process, or indeed, a forum shift, to develop a new pandemic treaty alongside the IHR.^35^ This proposal was followed by a call to action to spur moves for a new instrument or convention to enhance pandemic preparedness.^36^^,^^37^ A special session of the World Health Assembly was therefore held in 2021, and Member States agreed to establish an intergovernmental negotiating body to start the process of negotiating an international instrument, convention or other agreement. The EU favours a new instrument, as it supports international order based on well-functioning international institutions and a rule-based approach to global issues.^38^ Key actors such as China, the Russian Federation and the USA did not engage in the initial dialogue on a pandemic treaty.^36^^,^^39^ The fact that both the IHR amendments and the pandemic treaty are occurring under the auspices of WHO may create strategic differences between various governance mechanisms.

In September 2023, the High-Level Meeting on Pandemic Preparedness and Response took place during the UN General Assembly.^40^ This meeting was spurred by the Independent Panel for Pandemic Preparedness and Response,^41^ which noted that the UN was the appropriate forum to bring about meaningful change at the global level, given the success of interventions within the UN for the Ebola virus disease outbreaks.^42^ This high-level meeting was led by Israel and Morocco, with the support of some countries in the Group of 20 (G20) states that may feel without a voice within the working group for the IHR and intergovernmental negotiating body. In these latter forums, geopolitical negotiating relating to access to medicines tends to dominate between a few high-income states and the main counter-narrative of the so-called group for equity. For these states, the UN is the appropriate forum for pandemic preparedness, as it is where the heads of states meet – that is, those with political power to bring about change – and indeed, is a forum where they have had successes in other governance settings.^43^ WHO’s governing body is the World Health Assembly, which is generally composed of health ministers with more limited mandates.^41^ The text of the political declaration on pandemic prevention, preparedness and response^40^ bears considerable resemblance in content to the output of the intergovernmental negotiating body and working group for the IHR processes, yet the motivation for some forum shifting was to secure greater political support for pandemic preparedness and response.

Forum shifting is also occurring within global health security related to access to medical countermeasures, such as vaccines, which was arguably the biggest failure of multilateralism during COVID-19, given the considerable vaccine inequality during the pandemic.^44^ As such, the issue of countermeasures policy was always going to be central to any future policy development for global health security. The issue of medical countermeasures has been addressed in the various drafts of the IHR amendments, the intergovernmental negotiating body drafts, and the UN and G20 political declarations. These drafts have focused on equity to redress some of the inequalities experienced during the pandemic, with some proposals counteracting the status quo. Such policy development is particularly important within UN system forums, given the collective influence of low- and middle-income states in a one-state one-vote mechanism. Moreover, the aforementioned proliferation of forums where parallel conversations are occurring within the same system highlights the real need for low- and middle-income states to ensure their meaningful input into policy and operational content, to assure equity in access to and delivery of countermeasures in future.^45^ Additionally, this proliferation may suggest that some states are inclined to create strategic incoherence to avoid commitment to global equity in access to medical countermeasures. 

At the same time, forum shifting is occurring in another complex regime, the trade regime. High-income states recognize that debate on equity does not necessarily align with the domestic pressures they face to deliver countermeasures to their own populations, or maintain their relationship with the pharmaceutical industry. They would therefore prefer to keep the equity issue within the trade regime of WTO and the World Intellectual Property Organization (WIPO) as their enforcement mechanisms are more stringent than WHO.^46^^,^^47^ Maintaining this status quo would protect pharmaceutical patents and alleviate domestic pressures. Some G20 states led by Japan, and later India, have promoted a new institutional forum, the medical countermeasures platform, using the lessons learnt from the initiative COVID-19 Vaccines Global Access (COVAX) to develop an operational location to ensure research, access, technology transfer and equity.^48^ This forum seeks to offer an operational solution with regard to countermeasures, but it runs the risk of being perceived as being governed elsewhere and removed from the legal processes in Geneva within WHO, WTO and WIPO, potentially creating more disconnection between this platform and the legal developments arising from the working group for the IHR and the intergovernmental negotiating body. Given this situation, further forum shifting is occurring to regional mechanisms with, for example, the African Centres for Disease Control and Prevention becoming a new location for governance for African countries whose representatives feel that they gain little from global multilateral processes.^49^

Each of these processes claim to be improving global health security and indeed often indicate synergies with each other. Yet, when considering the global health security regime as a whole, it can be seen that proliferation of parallel activities and forum shifting is not uncoordinated or accidental, but reflects the political drivers of multilateralism.^50^ Indeed, as has been predicted,^11^ it would seem that a crisis of authority within and beyond WHO may be occurring. WHO is developing policy (for example, IHR amendments) at the request of Member States that recognize WHO as a comparatively non-political forum without strong political power to hold states to account, rather than the dominant actor.^51^ For WHO to develop policy when the decision-making authority has shifted to another forum, is futile, as is the case of access to medical countermeasures with the authority remaining with WTO. Thus, in terms of the regime complex, we are somewhere between a dominance of a concert of powers and a global rebalancing among states.^11^ The outcome of these ongoing processes will determine which of these scenarios emerges, and forum shifting remains a key mechanism by which states are trying to predetermine which occurs.

**Fig. 1:**
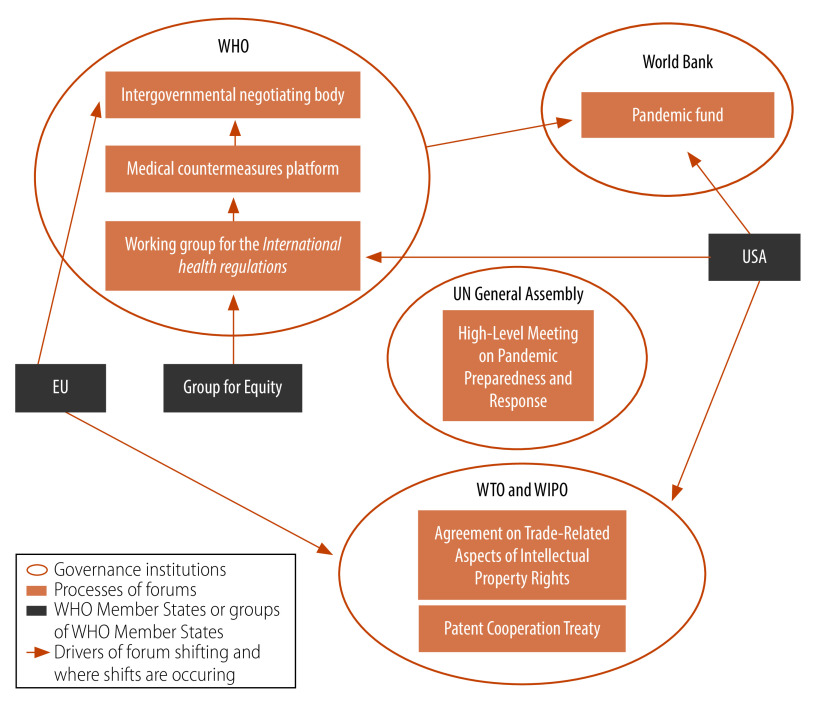
Contemporary forum shifting within the global health security regime

This forum shifting in global health security has secondary effects on WHO as an institution as it positions itself within the regime complex. Although WHO is the only multilateral institution mandated to be the international coordinating actor in global health, the potential forum shifting away from the Organization and the strategic uncertainty within it may be problematic, both for WHO and the global health security regime (Fig. 1).^22^ Practically, the forum shift away from WHO is at odds with the push governments are making for a strengthened WHO.^40^^,^^52^ Interestingly, despite being a technical organization, WHO exerts influence in these political processes and has moved within the changing forums to involve itself, such as acting as the technical lead for the Pandemic fund, and the governance of the intergovernmental negotiating body being located within the Organization. WHO is also the interim host of the medical countermeasures platform. The global health security regime complex may be placing WHO at the centre given its normative expertise as the directing and coordinating authority for international health work, to strengthen new institutions and forums. On the other hand, WHO may be accommodating forum shifting to maintain its position at the centre of the global health security regime. In the regime complex, stakeholders must act alongside each other and collaborate to maintain power and legitimacy, and deliver on their own activities.

EU European Union; UN: United Nations; WHO: World Health Organization; WIPO: World Intellectual Property Organization; WTO: World Trade Organization.

Understanding the regime complex and forum shifting is vital to the analysis of global health security and the inequalities that continue to beset negotiation processes and policy outcomes. To engage with the multiple current processes requires analysis of the factors that influence forum selection, including power dynamics, agenda-setting processes and strategic considerations. Additionally, forum shifting affects both the effectiveness of any one process and the legitimacy of global governance for health security at large, and for WHO in particular. This understanding can help public health practitioners and policy-makers develop strategies to engage politically in such processes, recognizing the political issues in play, to enhance coordination and coherence of efforts where possible and ultimately improve global health security.
